# THE ASSESSMENT OF VIEWS ON AGING: A REVIEW OF SELF-REPORT MEASURES AND INNOVATIVE EXTENSIONS

**DOI:** 10.1093/geroni/igz038.2896

**Published:** 2019-11-08

**Authors:** Nanna Notthoff, Ann-Kristin Beyer, Anne Blawert, Martina Gabrian, Verena Klusmann

**Affiliations:** 1 Leipzig University, Leipzig, Germany; 2 Charité Universitätsmedizin Berlin, Berlin, Berlin, Germany; 3 Friedrich-Alexander University of Erlangen-Nürnberg, Nuremberg, Bayern, Germany; 4 Independent Researcher Scientific Network Images of Aging, Frankfurt, Hessen, Germany; 5 University of Konstanz, Konstanz, Baden-Wurttemberg, Germany

## Abstract

Individual views on aging refer to either older people in general or to a person’s own age and aging. Classical approaches seem to hardly map the multidimensional, multidirectional, and highly individual nature of experiences as well as the malleability of perceptions of aging. For this paper, we reviewed existing measures of views of aging. These were categorized based on eight dimensions which were defined by an expert panel and based on current characterizations of views on aging in the literature. Results on 96 instruments call for a strengthening of the affective and the behavioral components in contrast to the apparently cognitive focus of the measures and argue for a stronger emphasis on the developmental nature of views on aging (time references, changes). This is particularly important when aiming to study the lifelong dynamics of views on aging. The suitability of innovative extensions will be discussed.

